# Antioxidative and Oxidative Profiles in Plasma and Saliva in Dairy Cows during Pregnancy

**DOI:** 10.3390/ani11113204

**Published:** 2021-11-10

**Authors:** Arletta Bielecka, Monika Jamioł, Marta Kankofer

**Affiliations:** Department of Biochemistry, Faculty of Veterinary Medicine, University of Life Sciences in Lublin, 20-033 Lublin, Poland; arletta.bielecka@up.lublin.pl (A.B.); monika.jamiol@up.lublin.pl (M.J.)

**Keywords:** oxidative stress, free radicals, antioxidants, pregnancy, cows

## Abstract

**Simple Summary:**

Pregnancy is a period of increased metabolic processes, which can lead to the formation of oxidative stress. The objective of the study was to investigate the influence of the altered metabolic state on the effectiveness of the antioxidant profile of plasma and saliva during the pregnancy of cows. In addition, we aimed to compare these biological fluids concerning their usefulness as possible markers of the physiological course of pregnancy. The results showed dynamic changes depending on the period of pregnancy course and revealed that the increase in oxidative intensity induced an appropriate answer of the organism. However, taking into account examined antioxidant/oxidative parameters, saliva reflects the content of plasma only in part, due to the local metabolism of the salivary gland. Therefore, further studies are necessary to establish physiological ranges of antioxidative/oxidative profiles of body fluids in cows.

**Abstract:**

Increased metabolism that occurs during pregnancy can result in oxidative stress which is harmful to cells and, consequently, for the proper functioning of the whole organism. Plasma and recently also saliva are important resources for evaluating physiological and pathological conditions in animals. The study aimed to investigate the influence of the metabolic state on the effectiveness of the antioxidant profile of plasma and saliva during the pregnancy of cows. Seventy-six healthy pregnant and twelve non-pregnant control cows were included in the study. Blood and saliva samples were collected each month of the pregnancy course. Examined body fluids were used to evaluate both the total antioxidant capacity (TAC) and the oxidative parameters related to protein and lipid peroxidative processes. TAC, the content of hydroperoxides, and SH groups were determined spectrophotometrically while formylokinurenine and bityrosine contents were measured spectrofluorimetrically. The results showed dynamic changes depending on the period of pregnancy course. The highest antioxidant activity in plasma was mostly noted in early pregnancy and advanced pregnant cows. All tested parameters except SH groups expressed higher values in saliva compared to plasma. Obtained results reveal that the increase in oxidative intensity induced appropriate answers of cells reflected in the increase in antioxidative activity of the organism. Moreover, some examined parameters can indicate the intensity of oxidative stress and therefore could be used in a panel of markers of the physiological course of pregnancy. However, with regards to antioxidant/oxidative parameters, saliva reflects the content of plasma only in part, due to the local metabolism of the salivary gland. Further studies are necessary to establish physiological ranges of antioxidative/oxidative profiles in cows and to define the usefulness of saliva as biological material in oxidative stress diagnostics.

## 1. Introduction

Reactive oxygen species (ROS) are intermediates that are formed during metabolic changes. They are constantly produced in the body and in small quantities are involved in the regulation of some physiological processes [1]. The well-known ROS that are generated as by-products of metabolism by biological systems are superoxide radicals (O_2_•^−^), hydrogen peroxide (H_2_O_2_), hydroxyl radicals (•OH), and singlet oxygen (^1^O_2_) [2]. ROS are mainly produced by mitochondria, both under physiological and pathological states. O_2_ may be produced by cellular respiration, lipoxygenase, and cyclooxygenase in the metabolism of arachidonic acid, endothelial cells, and inflammatory cells [3].

The balance between ROS formation and neutralization is controlled by the antioxidant systems of the body [4]. In a state of physiological equilibrium, the body has sufficient reserves of antioxidants necessary to neutralize free radicals. Enzymatic and non-enzymatic defense mechanisms neutralize ROS through various biochemical reactions [4,5]. Cells use an antioxidant defense system primarily based on enzyme components such as superoxide dismutase (SOD), catalase (CAT), and glutathione peroxidase (GPx) to protect against ROS-induced cellular damage [6]. Moreover, non-enzymatic scavengers are also important—glutathione, retinoids, carotenoids, ascorbic acid, vitamin E—as well as estrogens. Additionally, other equally significant molecules in the total antioxidative capacity of blood plasma are proteins. Albumins that are considered to be the major circulating non-enzymatic antioxidants prevent the production of free radicals by binding free metal ions [7].

An imbalance between redox processes causes excess ROS to lead to peroxidative damage to macromolecules not only in the cell but also in the cell membrane. This condition is known as oxidative stress [4,8]. As a consequence, the metabolic pathways are altered and the products of peroxidation have harmful effects even beyond the site of damage. This can happen when the organism is exposed to exogenous oxidizing agents or as a result of an insufficient antioxidant system that cannot effectively remove excess ROS or by breaking chain reactions [8,9]. Oxidative damage and ROS have been suggested to play a role in the initiation or development of many disorders and pathological conditions in livestock farms such as sepsis, enteritis, pneumonia, and arthritis, as well as reproductive disorders including udder edema, retention of fetal membranes, and mastitis [10].

During periods of increased metabolic activity such as pregnancy, there may be an imbalance between the production and accumulation of ROS in cells and tissues, and the ability of the biological system to detoxify these reactive products may occur [11,12]. The pregnancy period is related to extensive changes in protein synthesis in the uterus where the fetus is developed. These changes concern the remodeling of connective tissue and its proteins [13,14]. Any peroxidative damage of both biologically active or structural proteins may lead to pregnancy alterations and complications [15]. It is possible because free amino acids and amino acid residues in proteins are highly susceptible to oxidation by one or more reactive species. Protein oxidation processes lead to the modification of amino acid residues, aggregation resulting from cross-linking, fragmentation, and an increase in sensitivity of modified proteins to proteolysis [16,17]. 

There is evidence that during the periparturient period in cows the imbalance between ROS production and inactivation occurs. Kankofer and colleagues [8] demonstrated the presence of dynamic changes of the antioxidative/oxidative profiles during the periparturient period in the plasma of cows with indications of oxidative stress during the final two weeks of pregnancy. Similar changes associated with late pregnancy were observed by Castillo and colleagues [18]. The profile of antioxidative/oxidative balance during whole pregnancy is absent in the available literature. 

Parameters of antioxidative and prooxidative profile can be detected in blood plasma and also in saliva which is a biological fluid of special importance as it is collected in a non-invasive way [19]. Whether saliva also reflects metabolic profile remains unclear due to the dynamics and complexity of the saliva proteome [20]. 

The hypothesis was stated that pregnancy is a period of increased metabolic processes, which can lead to the formation of oxidative stress. Moreover, the feasibility of using saliva as diagnostic material as an alternative to blood to assess changes in antioxidant status and oxidative stress parameters can be examined. 

This study aimed to investigate the impact of the altered metabolic state on the effectiveness of the antioxidant profile to defend against ROS during pregnancy in the plasma and saliva of cows. An additional aim was to compare these two biological fluids with regard to their usefulness as possible markers of the physiological course of pregnancy.

## 2. Materials and Methods

Plasma and saliva were collected during routine veterinary procedures in accordance with the principles of antiseptics from clinically healthy, sexually mature non-pregnant (aged 2–8 years; n = 12), and pregnant Polish Holstein dairy cows (aged 2–8 years; n = 76, 1–9 months pregnant). The cows were from the same farm. The average milk yield was 9000 L per cow per year. All examined cows were sampled once.

From each animal, blood was collected at a similar time of day from the coccygeal vein via puncture, into tubes with anticoagulant and centrifuged at 1500 rpm for 15 min at 4 °C. Plasma samples were portioned and stored at −20 °C until they were used for analysis. 

Saliva was collected at a similar time of day with sponges mounted in the space between the teeth and cheeks. Materials were then centrifuged at 1500 rpm for 15 min at 4 °C, portioned, and frozen at −20 °C until they were tested.

### 2.1. Total Antioxidant Capacity 

The concentration of total antioxidant capacity (TAC) was measured according to the method of Benzie and Strain (1996) [21], based on the ferric reducing ability of plasma (FRAP) with some modifications. The working reagent contained 300 mmol/L acetate buffer (pH 3.6), 10 mmol/L 2,4,6 tri pyridyl striazine (TPTZ, Sigma, Poznań, Poland) solution in 40 mmol/L HCl, and 20 mmol/L FeCl_3_ × 6H_2_O solution in distilled H_2_O, mixed to the ratio of 10:1:1. The mixture was prepared immediately before use. The working reagent (2250 μL) was mixed with 25 μL of sample and absorbance was measured at 593 nm. The working reagent itself was used as a control. After 10 min of incubation at room temperature, the absorbance was checked again. The difference in absorbance at 0 and at 10 min was compared with a standard curve prepared with 10 different dilutions of Fe (II) between 0 and 1000 μmol/L. The results were expressed as μmol/g protein. Each determination was performed in duplicate.

Changes in absorbance were directly related to the ‘total’ reducing capacity of the electron-donating antioxidants that were present in the examined plasma and saliva.

### 2.2. The Content of Hydroperoxides

The content of hydroperoxides was measured according to the method of Alberti et al. (2000) [22]. A volume of 20 μL blood plasma or saliva was diluted in 1 mL of 100 mmol/L acetate buffer (pH 4.8) and 10 μL 3.7 × 10^−1^ M of an aqueous solution of N,N,diethyl para-phenylenediamine (DEPPD) was added. After 90 min of incubation at 37 °C, absorbances were read at 505 nm against acetate buffer alone. In the control sample, 20 μL of distilled water was added instead of sample. The results were expressed as μmol/g protein. Each determination was performed in duplicate.

### 2.3. The Content of Sulfhydryl Groups 

The concentrations of sulfhydryl residues (SH) in plasma and saliva were measured by spectrophotometry, as detailed by Rice Evans and colleagues (1991) [23]. A volume of 300 μL 10% (*w*/*v*) sodium dodecyl sulphate (SDS, Sigma, Poznań, Poland) in 10 mmol/L sodium phosphate buffer (pH 8.0) was added to 300 μL of sample and mixed. A 2.4 mL of 10 mmol/L sodium phosphate buffer (pH 8.0) was added. Then, 300 μL 20 mg of 5,5-dithiobis- 2-nitro benzoate (Sigma, Poznań, Poland) in 50 mL of buffer (DTNB) was added and the absorbance was measured at 412 nm. The control sample contained 300 μL of the same buffer. All samples were incubated for 1 h at 37 °C. After incubation, the absorbance was measured again at 412 nm. The difference between absorbance before and after incubation (after subtracting the respective absorbance of the control) pointed to the content of SH groups. The content was calculated using a standard curve prepared with different dilutions of glutathione (GSH, Sigma, Poznań, Poland) ranging from 0 to 1 mmol/L in 10 mmol/L sodium phosphate buffer (pH 8.0) and was expressed in mmol/g protein. Each determination was performed in duplicate.

### 2.4. The Content of Bityrosine Bridges 

Bityrosine bridges were determined by a spectrofluorimetric method according to Rice Evans and colleagues (1991) [23]. The diluted plasma and saliva samples were excited by light at 325 nm and emission was measured at a wavelength of 410 mm. The spectrofluorometer was standardized to 100 deflections with chinine sulphate (0.1 μg/ mL in 0.1 mol/ H₂SO₄) at excitation (350 nm) and emission wavelength (445 nm). The results were expressed as μg/mg protein. Each determination was performed in duplicate.

### 2.5. The Content of Formylokinurenine 

Formylokinurenine was determined by a spectrofluorimetric method described by Rice Evans and colleagues (1991) [23]. The diluted plasma and saliva samples were excited by light at 360 nm and emission was measured at a 454 nm wavelength. The spectrofluorimeter (Jasco, Tokyo, Japan) was standardized, as described above. The results were expressed as μg/mg protein. Each determination was performed in duplicate.

### 2.6. Protein Content 

The protein content of the plasma samples was measured by the biuret method, using commercial assay kits (Cormay, Łomianki, Poland) based on the method reported by Gornal and colleagues (1949) [24]. 

The protein content of the saliva samples was measured according to the Bradford method [25] using commercial reagents (Bradford Reagent, Sigma, Poznań, Poland).

### 2.7. Statistical Analysis

Individual data in duplicate were subjected to normality test (Shapiro–Wilk test) [26] and equal variance test (Levene’s test) [27]. Differences between groups were tested using Kruskal–Wallis [28] and Mann–Whitney *U* [29] tests. A *p*-value of <0.05 was considered statistically significant. The analysis was performed with STATISTICA Version 13.0 (StatSoft, Poland, TIBCO Software Inc., Palo Alto, CA, USA). Data are expressed as the means +/− standard deviation.

## 3. Results

**Figure 1:**
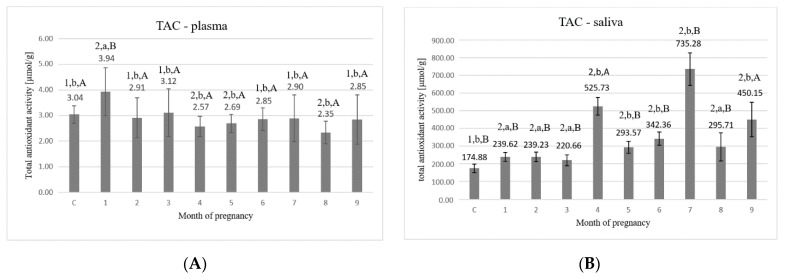
(**A**) Total antioxidant capacity (TAC) values in plasma; (**B**) total antioxidant capacity (TAC) values in saliva. C—control group, 1–9—months of pregnancy; 1, 2—different numbers above the bars mean significant differences (*p* < 0.05) between the control group and examined months of pregnancy; a,b—different small letters above the bars mean significant differences (*p* < 0.05) between 1st month and control group as well as examined months of pregnancy; A,B—different big letters above the bars mean significant differences (*p* < 0.05) between 9th month and control as well as examined months of pregnancy. Data are expressed as the means +/− standard deviation.

Analysis of the TAC in plasma (Figure 1A) showed that, among the 9 months of pregnancy, the highest values were observed in the first three months (3.94 ± 0.928 μmol/g, 2.91 ± 0.782 μmol/g, 3.12 ± 0.932 μmol/g, respectively). The control group reached the value of 3.04 ± 0.337 μmol/g protein and was significantly (*p* < 0.05) lower compared to the first month of pregnancy. In the next months of pregnancy (4th–7th), the values tended to increase from 2.57 ± 0.393 μmol/g up to 2.90 ± 0.913 μmol/g. In the 9th month of pregnancy, the value increased as compared to the 8th month and reached the value of 2.85 ± 0.962 μmol/g. The TAC value in the 1st month of pregnancy was the highest and statistically significantly higher than other examined time points.

Statistically significant differences (*p* < 0.05) were detected between the following time points: C–1, C–4, C–5, C–8, 1– all of the other months of pregnancy, 2–8, 3–4, 3–8, 5–8, 6–8, and 7–8.

The TAC values in saliva (Figure 1B) were significantly higher than those observed in the plasma of all tested samples when calculated relative to the protein content of each biological fluid. In the first three months, significantly lower values were detected compared to the later period of pregnancy, unlike in the case of TAC in plasma. The highest values in saliva were found in the 4th (525.73 ± 50.33 μmol/g), 7th (735.28 ± 91.77 μmol/g), and 9th month (450.15 ± 97.51 μmol/g) which were significantly increased than in control (*p* < 0.05). Non-pregnant cows are characterized by the lowest value of TAC as compared to pregnant cows. An increase in antioxidant activity was observed in the 9th month (450.15 ± 97.51 μmol/g), compared to 8th month (295.71 ± 79.06 μmol/g), as in the case of plasma.

Statistically significant differences (*p* < 0.05) were detected between the following time points: C–all of the other months of pregnancy, 1–4, 1–5, 1–6, 1–7, 1–9, 2–4, 2–5, 2–6, 2–7, 2–9, 3–4, 3–5, 3–6, 3–7, 3–9, 4–5, 4–6, 4–7, 4–8, 5–6, 5–7, 5–9, 6–7, 6–9, 7–8, 7–9, and 8–9.

**Figure 2:**
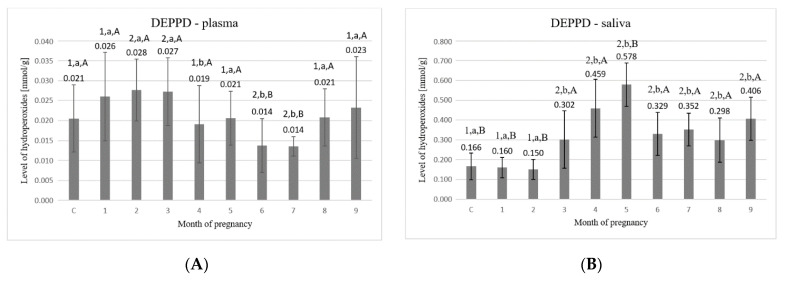
(**A**) Values of hydroperoxides (DEPPD) in plasma; (**B**) values of hydroperoxides (DEPPD) in saliva. C—control group, 1–9—months of pregnancy; 1, 2—different numbers above the bars mean significant differences (*p* < 0.05) between the control group and examined months of pregnancy; a,b—different small letters above the bars mean significant differences (*p* < 0.05) between 1st month and control group as well as examined months of pregnancy; A,B—different big letters above the bars mean significant differences (*p* < 0.05) between 9th month and control as well as examined months of pregnancy. Data are expressed as the means +/− standard deviation.

Analysis of the content of hydroperoxides in plasma (Figure 2A) showed that the highest values were observed in the 2nd and the 3rd month of pregnancy (0.028 ± 0.008 mmol/g and 0.027 ± 0.008 mmol/g, respectively), which were significantly higher than in control (*p* < 0.05). The control group reached the value of 0.021 ± 0.008 mmol/g protein. In the 6th and 7th months, the values decreased significantly (*p* < 0.05) as compared to the first three months and reached the value of 0.014 ± 0.007 mmol/g and 0.014 ± 0.002 mmol/g, respectively. From the 6th to 9th month of pregnancy, the values tended to increase from 0.014 ± 0.007 mmol/g to 0.023 ± 0.013 mmol/g.

Statistically significant differences (*p* < 0.05) were detected between the following time points: C–2, C–3, C–6, C–7, 1–4, 1–6, 1–7, 2–4, 2–6, 2–7, 3–4, 3–5, 3–6, 3–7, 3–8, 5–6, 5–7, 6–8, 6–9, 7–8, and 7–9.

Detected contents of hydroperoxides in saliva (Figure 2B) were significantly higher than in plasma, similar to in the case of TAC. Throughout the pregnancy, the significantly (*p* < 0.05) lowest values were found in the first two months (0.160 ± 0.052 mmol/g and 0.150 ± 0.051 mmol/g, respectively). In the subsequent months, there was a significant increase in the hydroperoxides content—from a value of 0.302 ± 0.145 mmol/g in the 3rd month to 0.578 ± 0.110 mmol/g in the 5th month. Observed hydroperoxides content from the 6th to the 8th month decreased and remained at a similar level of about ~0.3 mmol/g. The value in the 9th month increased compared to the 8th month, reaching a value of 0.406 ± 0.109 mmol/g.

Statistically significant differences (*p* < 0.05) were detected between the following time points: C–3, C–4, C–5, C–6, C–7, C–8, C–9, 1–3, 1–4, 1–5, 1–6, 1–7, 1–8, 1–9, 2–3, 2–4, 2–5, 2–6, 2–7, 2–8, 2–9, 3–5, 4–6, 4–7, 4–8, 5–6, 5–7, 5–8, and 5–9.

**Figure 3:**
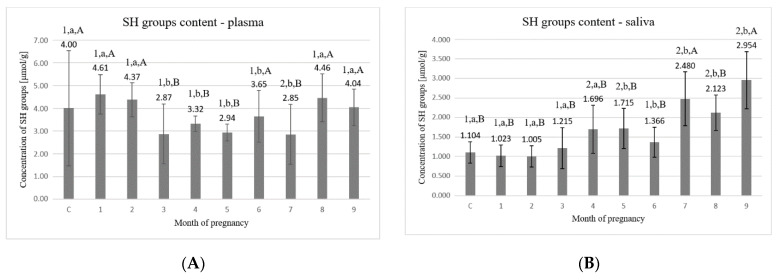
(**A**) Concentration of sulfhydryl groups (SH) in plasma; (**B**) concentration of sulfhydryl groups (SH) in saliva. C—control group, 1–9—months of pregnancy; 1,2—different numbers above the bars mean significant differences (*p* < 0.05) between the control group and examined months of pregnancy; a,b—different small letters above the bars mean significant differences (*p* < 0.05) between 1st month and control group as well as examined months of pregnancy; A,B—different big letters above the bars mean significant differences (*p* < 0.05) between 9th month and control as well as examined months of pregnancy. Data are expressed as the means +/− standard deviation.

The highest values of the concentration of SH groups in plasma (Figure 3A) were detected in the first two months (4.61 ± 0.862 μmol/g and 4.37 ± 0.742 μmol/g, respectively) and the last two months of pregnancy (4.46 ± 1.053 μmol/g and 4.04 ± 0.800 μmol/g, respectively). Compared to these months, significantly (*p* < 0.05) lower values were found in the period from the 3rd to the 7th month, where the values tender to fluctuate on the level of about ~3.0 µmol/g.

Statistically significant differences (*p* < 0.05) were detected between the following time points: C–7, 1–3, 1–4, 1–5, 1–6, 1–7, 2–3, 2–4, 2–5, 2–7, 3–8, 3–9, 4–8, 4–9, 5–8, 5–9, 6–8, 7–8, and 7–9.

The concentration of sulfhydryl groups in saliva (Figure 3B) slightly increased in the 4th–5th months compared to the period from the 1st to the 3rd month. Significantly higher values (*p* < 0.05) were noted at the end of the pregnancy period, from the 7th to the 9th month (2.480 ± 0.688 μmol/g, 2.123 ± 0.453 μmol/g, and 2.954 ± 0.729 μmol/g, respectively).

Statistically significant differences (*p* < 0.05) were detected between the following time points: C–4, C–5, C–7, C–8, C–9, 1–5, 1–6, 1–7, 1–8, 1–9, 2–4, 2–5, 2–6, 2–7, 2–8, 2–9, 3–5, 3–7, 3–8, 3–9, 4–7, 4–9, 5–6, 5–7, 5–8, 5–9, 6–7, 6–8, 6–9, and 8–9.

**Figure 4:**
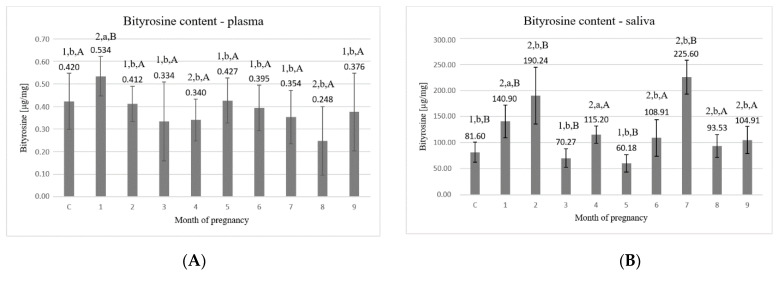
(**A**) Bityrosine content in plasma; (**B**) bityrosine content in saliva. C—control group, 1–9—months of pregnancy; 1,2—different numbers above the bars mean significant differences (*p* < 0.05) between the control group and examined months of pregnancy; a,b—different small letters above the bars mean significant differences (*p* < 0.05) between 1st month and control group as well as examined months of pregnancy; A,B—different big letters above the bars mean significant differences (*p* < 0.05) between 9th month and control as well as examined months of pregnancy. Data are expressed as the means +/− standard deviation.

The highest concentration of bityrosine bridges in the plasma (Figure 4A) was detected in the 1st month of pregnancy (0.534 ± 0.087 μg/mg) which was significantly higher than in control (*p* < 0.05). From the 2nd to the 7th month, values ranged between 0.334 ± 0.174 μg/mg and 0.427 ± 0.100 μg/mg. In the last two months of pregnancy (8th–9th), there was an increase in the concentration of bityrosine bridges from the value of 0.248 ± 0.152 μg/mg in the 8th month to the value of 0.376 ± 0.173 μg/mg in the 9th month.

Statistically significant differences (*p* < 0.05) were detected between the following time points: C–1, C–4, C–8, 1–all of the other months of pregnancy, 2–4, 2–8, 5–8, 6–8, and 7–8.

The concentrations of bityrosine bridges in saliva (Figure 4B) were significantly higher than those detected in plasma. The highest values were found in the 1st and the 2nd month (140.90 ± 31.39 μg/mg and 190.24 ± 54.35 μg/mg, respectively) and in the 7th month (225.60 ± 32.68 μg/mg), which were significantly higher than in control (*p* < 0.05). Months 8th–9th remained at a similar value and did not differ significantly from each other.

Statistically significant differences (*p* < 0.05) were detected between the following time points: C–1, C–2, C–4, C–6, C–7, C–8, C–9, 1–2, 1–3, 1–5, 1–6, 1–7, 1–8, 1–9, 2–3, 2–4, 2–5, 2–6, 2–8, 2–9, 3–4, 3–6, 3–7, 3–9, 4–5, 4–7, 5–6, 5–7, 5–8, 5–9, 6–7, 7–8, and 7–9.

**Figure 5:**
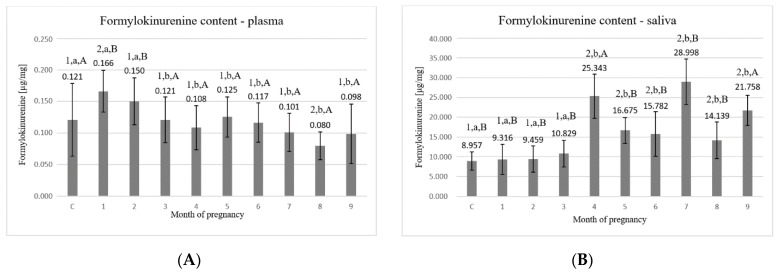
(**A**) Formylokinurenine content in plasma; (**B**) formylokinurenine content in saliva. C—control group, 1–9—months of pregnancy; 1,2—different numbers above the bars mean significant differences (*p* < 0.05) between the control group and examined months of pregnancy; a,b—different small letters above the bars mean significant differences (*p* < 0.05) between 1st month and control group as well as examined months of pregnancy; A,B—different big letters above the bars mean significant differences (*p* < 0.05) between 9th month and control as well as examined months of pregnancy. Data are expressed as the means +/− standard deviation.

The highest concentration of formylokinurenine in plasma (Figure 5A) was detected in the 1st and the 2nd month (0.166 ± 0.033 μg/mg and 0.150 ± 0.037 μg/mg, respectively). In the next months of pregnancy (3rd–9th), the values tender to fluctuate from 0.080 ± 0.022 μg/mg to 0.125 ± 0.032 μg/mg. The control group reached the value of 0.121 ± 0.058 μg/mg protein and was significantly (*p* < 0.05) different from the 1st (0.166 ± 0.033 μg/mg) and the 8th (0.080 ± 0.022 μg/mg) month of pregnancy. 

Statistically significant differences (*p* < 0.05) were detected between the following time points: C–1, C–8, 1–3, 1–4, 1–5, 1–6, 1–7, 1–8, 1–9, 2–3, 2–4, 2–5, 2–6, 2–7, 2–8, 2–9, 3–8, 4–8, 5–8, 6–8, and 7–8.

Formylokinurenine concentrations found in saliva (Figure 5B) were significantly higher than in plasma. At the beginning of pregnancy (month 1st–3rd), the values were the lowest compared to the remaining months. The highest values were obtained in the 4th (25.343 ± 5.608 μg/mg), in the 7th (28.998 ± 5.707 μg/mg), and in the 9th month (21.758 ± 3.842 μg/mg), which were significantly increased compared to the control (*p* < 0.05). In both saliva and plasma, a similar relationship was noted at the end of pregnancy. Compared to the 8th month (14.139 ± 4.623 μg/mg), in the 9th month, there was an increase in the formylokinurenine concentration to the value of 21.758 ± 3.842 μg/mg.

Statistically significant differences (*p* < 0.05) were detected between the following time points: C–4, C–5, C–6, C–7, C–8, C–9, 1–4, 1–5, 1–6, 1–7, 1–8, 1–9, 2–4, 2–5, 2–6, 2–7, 2–8, 2–9, 3–4, 3–5, 3–7, 3–9, 4–5, 4–6, 4–7, 4–8, 5–7, 5–9, 6–7, 6–9, 7–8, 7–9, and 8–9.

Protein concentration in plasma was approx. 76.14 g/L, in saliva –1.17 g/L.

## 4. Discussion

This study presents the relationship between the profile of the antioxidant capacity and oxidative intensity of the plasma and saliva collected from pregnant cows in each month of pregnancy and pregnancy course. The results showed differences depending on the period of pregnancy. These differences may indicate a possible role of the antioxidant system of the organism, which is altered during pregnancy. The observed fluctuations of antioxidant defense of organisms could be related to changes in metabolism intensity based on the need for increased protein synthesis by the mother associated with fetal growth and development [30].

ROS are constantly generated in the body and they are involved in many metabolic processes [1]. Excessive production of ROS or the reduced efficiency of antioxidants leads to oxidative stress and consequently to various pathological conditions, such as peroxidative damage of tissues, cells, and biologically active molecules [12,31]. These changes may lead to disturbances in metabolic pathways and clinical symptoms of illness, e.g., retained placenta in cows [32]. Furthermore, physiological pregnancy as a period of increased metabolic activity may cause an imbalance between the production and neutralization of ROS [11,12].

Saliva is a body fluid containing electrolytes, hormones, proteins, and other organic compounds produced mostly by salivary glands with a small portion originating from the blood [33]. This makes saliva a potential source of information about the physiological status of animals, including animal diseases. Moreover, the advantage of saliva is that it can be easily obtained in a non-invasive way, which is relatively stress free for animals [34]. For these reasons, the use of saliva as a diagnostic material is of great interest to scientists.

TAC values represent the total capacity of the organism to defend ROS and cover substances of different chemical structures. Our results showed some differences within this parameter between the months of pregnancy both in plasma and saliva. Interestingly, the TAC of saliva was higher than the TAC of plasma which may indicate a more powerful saliva solution. The lowest levels of the TAC in saliva were found at the beginning of pregnancy (in contrast to plasma levels). It can be concluded that the changes in TAC capacity in saliva are secondary to plasma. Moreover, cows adjust their antioxidative defense to their needs related to particular stages of pregnancy such as implantation, placentation, and parturition. These stages represent special metabolic activities that occur within the course of pregnancy and are related to mother–fetus interaction, placenta development, and pregnancy development.

Another side effect of reactions taking place through the presence of free radicals is the degradation of the cell membranes. Frequent targets of peroxy radicals are polyunsaturated fatty acids, which, due to the presence of a methylene group between two double bonds, are characterized by easily removable hydrogens [35]. The indirect method used here is concerned with the monitoring of a rather persistent radical cation formed in the reaction of alkoxy and peroxy radicals derived from the hydroperoxides with a suitable additive, i.e., DEPPD. In the present study, lipid peroxidation in plasma was significantly higher in early pregnancy and advanced pregnant cows. This is in agreement with the TAC determinations showing the appropriate answer of the organism to the increase in oxidative intensity. The findings of our study are in corroboration with the reports of Gaál et al. (2006) [36].

SH groups, which are present in cysteine and the vast majority of peptides and proteins, are highly susceptible to ROS attack [37]. Cysteine is an amino acid that, due to the presence of the SH group in its structure, most often undergoes oxidation, resulting in the formation of disulfide bridges. Any modifications of protein structure cause changes in or loss of their biological activity, changes in the functions or inactivation of enzymes, as well as affect the ability to bind receptors or other biologically active substances [38]. The content of cysteine SH groups as redox sensors can be used as a measure of protein peroxidation intensity since any decrease in these levels may indicate peroxidative damage to proteins [8,39]. This study showed relatively higher concentrations of SH groups at the end of pregnancy which may point to the regeneration of protein molecules or their synthesis which is related to the development of the fetus as well as the development of the uterine environment assuring appropriate growth of the fetus. 

Proteins are highly sensitive to the action of free radicals [37]. Other amino acids susceptible for oxidation are aromatic amino acids (tryptophan, tyrosine, phenylalanine), and their modification results in formylokinurenines, kinurenines (from tryptophan), and bis-tyrosyl bridges (from phenylalanine and tyrosine) [30,40].

The concentrations of formylokinurenine and bityrosine bridges were significantly higher in the saliva than in the plasma of the examined animals. Moreover, recorded concentrations were rather higher in the first two months and the last month of pregnancy. This may confirm the increase in metabolic activity related to the course of pregnancy and possible alterations between antioxidative and oxidative balance [41,42]. Interestingly, the increased values of concentrations of formylokinurenine and bityrosine bridges in saliva were recorded in the 4th and 7th month and the increased values of TAC in these months were probably a consequence of it.

All parameters examined here should not be considered separately as they depend on each other, and this relationship is visible also in the present paper—the increase in peroxidative intensity is reflected in the adequate antioxidative answer. Our team confirmed earlier experiments of Castillo et al. (2005) who confirmed the characteristic metabolic changes associated with late pregnancy and early lactation as well as an increased lipid peroxidation around the parturition period [18]. Our team examined early-mid pregnancy and parturient status of plasma and confirmed relationships between the course of pregnancy and antioxidative/prooxidative profile [8,41]. As long as this relationship exists and an answer occurs the cells and tissues can respond physiologically and avoid serious peroxidative damage. The lack of reaction to the increase in peroxidative activity may lead to peroxidative damage and serious biochemical consequences [15,43,44]. 

The reason for the possible imbalance in the early pregnancy period could be related to the rise in oxygen tension which is associated with the formation of placental circulation [45]. This status accompanies the increase in the expression of antioxidative enzymes in the placenta as well as the increase in tissue oxygen requirements [46]. As a consequence, oxidation of SH groups may occur.

It is possible, to some extent, to control and influence the relationship between antioxidative and oxidative balance by feeding [11,47]. This statement was also confirmed by the determinations of antioxidative/oxidative profile in the same cows in two consecutive lactations [48] where two consecutive years expressed differences between samples.

Whether pregnancy can influence the antioxidative/pro-oxidative status of non-pregnant and pregnant animals, experiments were carried out on cows and bitches [49,50,51]. Authors observed significant differences between physiological status in examined parameters and related the observed changes to the hormonal profile, hormonal control of antioxidative enzymes as well as estrogens itself as ROS scavengers. Moreover, higher values of TAC in saliva as in plasma were confirmed.

The present paper is part of a project on searching for markers of physiological pregnancy and parturition. The results obtained here reveal that some parameters can be used in a panel of markers of the proper course of pregnancy while pointing to metabolic changes in the organisms, as well as antioxidative/oxidative profile.

## 5. Conclusions

In summary, the present study revealed changes in the antioxidative/oxidative profiles of plasma and saliva during the pregnancy course in cows. In corroboration with other studies, recorded dynamic changes were observed in early pregnancy and advanced pregnant cows that can be related to the implantation, placentation, and parturition phase. However, with regards to antioxidant/oxidative parameters, saliva reflects the content of plasma only in part, due to the local metabolism of the salivary gland. The study is limited by the lack of other parameters related to the course of pregnancy. These parameters would have allowed us to link the tested data of saliva and blood plasma. Although no other characteristic for the pregnancy parameters has been identified, it is clear that the course of pregnancy should be associated with a well-known hormonal profile. Additionally, to limit the individual variability, the same cow could be sampled each month of the pregnancy in a repeated measures design.

Further studies are necessary to establish physiological ranges of antioxidative/oxidative profiles in cows and to define the usefulness of saliva as biological material in the estimation of this profile.

## Data Availability

Not applicable.
